# Supplementary material for: Effectiveness of Snail Slime in the Green Synthesis of Silver Nanoparticles
Source: Nanomaterials (Basel). 2022 Oct 1;12(19):3447. doi: 10.3390/nano12193447 (PMC9565232; doi:10.3390/nano12193447)
Supplement: Supplementary file 1 [file nanomaterials-12-03447-s001.zip › nanomaterials-1931594-supplementary.pdf]

Supplemenary Materials

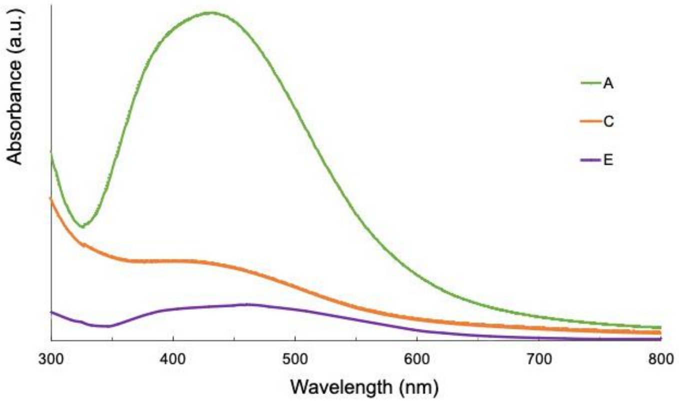

Figure S1. UV-visible spectra collected from samples A,C and E.

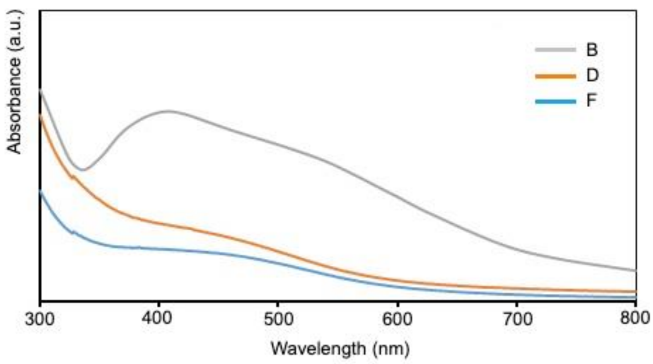

Figure S2. UV-visible spectra collected from samples B, D and F.

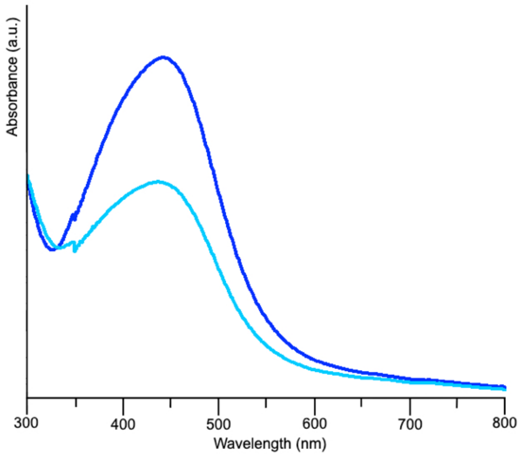

Figure S3. UV-vis spectra recorded from sample H after 3 (light blue line) and 5 days (blue line) from mixing.

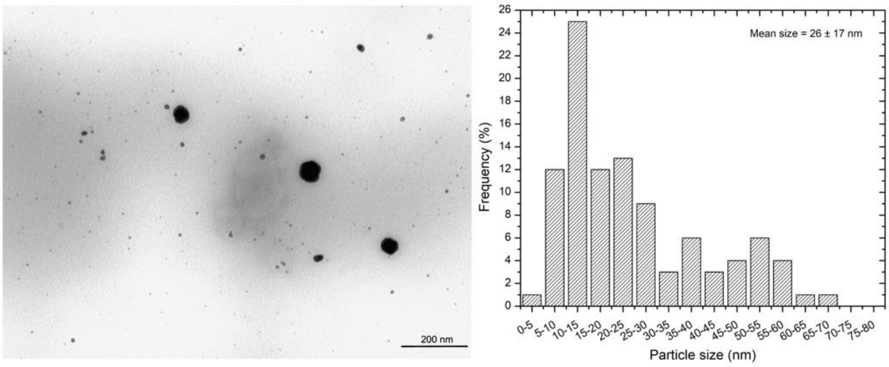

Figure S4. TEM image (left) and evaluation of size distribution (right) of AgNPs_Ref. Scale bar = 200 nm.

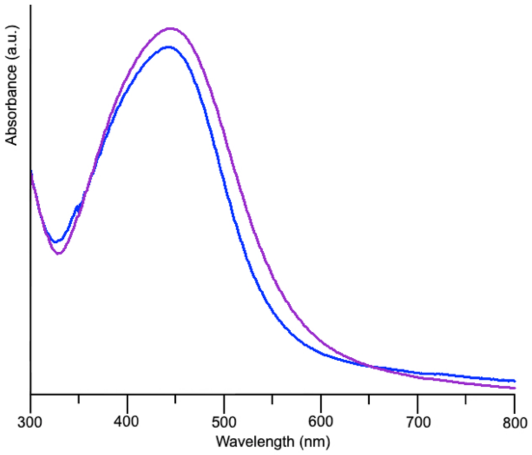

Figure S5. UV-vis spectra of AgNPs obtained from synthesis H collected after five days from the mixing of the reactants (blue line) and after fifty days (purple line).

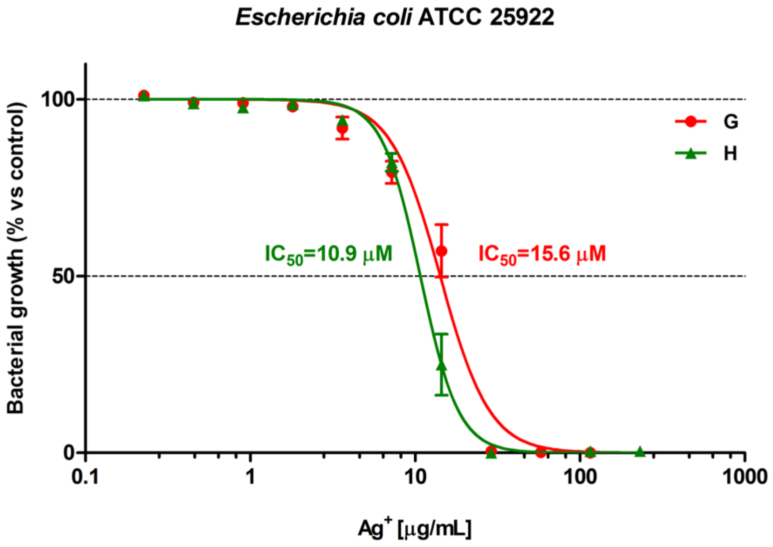

Figure S6. Dose-response curves for samples G and H obtained for E. coli.

Table S1. One-way analysis of variance (ANOVA) of IC_{50} values obtained for *S. aureus* and *E. coli*.

Table Analyzed	*S. aureus*
One-way analysis of variance	

P value	0,7838				
P value summary	ns				
**Are means signif. different? (P < 0.05)**	**No**				
Number of groups	4				
F	0,3610				
R squared	0,1529				
ANOVA Table	SS	df	MS		
Treatment (between columns)	25400	3	8467		
Residual (within columns)	140700	6	23460		
Total	166100	9			
**Tukey's Multiple Comparison Test**	Mean Diff.	q	Significant? P < 0.05?	Summary	95% CI of diff
AgNO3 vs G	-79,68	0,8060	No	ns	-563.7 to 404.3
AgNO3 vs H	-10,40	0,09603	No	ns	-540.6 to 519.8
AgNO3 vs Reference	-123,8	1,252	No	ns	-607.8 to 360.2
G vs H	69,28	0,7008	No	ns	-414.7 to 553.3
G vs Reference	-44,13	0,4991	No	ns	-477.1 to 388.8
H vs Reference	-113,4	1,147	No	ns	-597.4 to 370.6

**Table Analyzed**	***E. coli***				
**One-way analysis of variance**					
P value	0,8967				
P value summary	ns				
**Are means signif. different? (P < 0.05)**	**No**				
Number of groups	5				
F	0,2645				
R squared	0,05858				
ANOVA Table	SS	df	MS		
Treatment (between columns)	54,30	4	13,58		
Residual (within columns)	872,7	17	51,33		
Total	927,0	21			
**Tukey's Multiple Comparison Test**	Mean Diff.	q	Significant? P < 0.05?	Summary	95% CI of diff
AgNO3 vs A	2,248	0,6076	No	ns	-13.67 to 18.17
AgNO3 vs G	-0,1213	0,03954	No	ns	-13.32 to 13.08
AgNO3 vs H	4,051	1,192	No	ns	-10.57 to 18.67
AgNO3 vs Reference	1,744	0,5132	No	ns	-12.88 to 16.37
A vs G	-2,369	0,6613	No	ns	-17.78 to 13.05
A vs H	1,803	0,4659	No	ns	-14.85 to 18.45
A vs Reference	-0,5037	0,1302	No	ns	-17.15 to 16.15
G vs H	4,172	1,276	No	ns	-9.900 to 18.24
G vs Reference	1,865	0,5704	No	ns	-12.21 to 15.94
H vs Reference	-2,307	0,6438	No	ns	-17.72 to 13.11
